# Leaf Surface Lipophilic Compounds as One of the Factors of Silver Birch Chemical Defense against Larvae of Gypsy Moth

**DOI:** 10.1371/journal.pone.0121917

**Published:** 2015-03-27

**Authors:** Vyacheslav V. Martemyanov, Sergey V. Pavlushin, Ivan M. Dubovskiy, Irina A. Belousova, Yuliya V. Yushkova, Sergey V. Morosov, Elena I. Chernyak, Victor V. Glupov

**Affiliations:** 1 Laboratory of Insect Pathology, Institute of Systematics and Ecology of Animals SB RAS, 630091, Frunze str. 11, Novosibirsk, Russia; 2 Laboratory of Ecological Research and Chromatographic Analysis, Novosibirsk Institute of Organic Chemistry SB RAS, 630090, Lavrentjev Ave. 9, Novosibirsk, Russia; Natural Resources Canada, CANADA

## Abstract

Plant chemical defense against herbivores is a complex process which involves a number of secondary compounds. It is known that the concentration of leaf surface lipophilic compounds (SLCs), particularly those of flavonoid aglycones are increased with the defoliation treatment of silver birch *Betula pendula*. In this study we investigated how the alteration of SLCs concentration in the food affects the fitness and innate immunity of the gypsy moth *Lymantria dispar*. We found that a low SLCs concentrations in consumed leaves led to a rapid larval development and increased females’ pupae weight (= fecundity) compared to larvae fed with leaves with high SLCs content. Inversely, increasing the compounds concentration in an artificial diet produced the reverse effects: decreases in both larval weight and larval survival. Low SLCs concentrations in tree leaves differently affected larval innate immunity parameters. For both sexes, total hemocytes count in the hemolymph increased, while the activity of plasma phenoloxidase decreased when larvae consume leaves with reduced content of SLCs. Our results clearly demonstrate that the concentration of SLCs in silver birch leaves affects not only gypsy moth fitness but also their innate immune status which might alter the potential resistance of insects against infections and/or parasitoids.

## Introduction

Insects play an important role in the evolution of plants, which offer defense mechanisms against insects under herbivore pressure to save their structural and functional continuity [1]. Plant adaptations that lead to decreased herbivore damage are termed as plant resistance. Among the mechanisms of plants resistance, researchers distinguish between physical and chemical barriers [2–3]. The former includes the thickness of the cuticle of a leaf, its waxy layer, the presence of leaf hairiness (trichomes) and other physical attributes. The latter includes the content of both primary (mainly proteins and carbohydrates) and secondary (phenols, terpenoids, alkaloids, etc.) compounds. The content of the nutrients in leaves directly influences the accumulation of energy and mass by insects during ontogenesis. Secondary metabolites can have a repellent effect on insects, reducing their preferences for plants [4] and even display a direct toxic/antinutritive effect [4–5].

One important category of allelochemicals involved in the chemical defense of plants against folivorous insects are the lipophilic compounds present on the leaf surface [6–10]. Leaf surface lipophilic compounds (SLCs) of deciduous trees include the following allelochemicals: hydrocarbons, terpenoids, flavonoid aglycones, fatty acids and some other compounds [9–10]. These lipophilic compounds are secreted and accumulated within glandular trichomes—projections from protodermal cells [11]. The toxicity of these compounds depends on both plant species and plant attacker species (i.e. phytopathogens and herbivores) [9]. Their defensive role against a leaf-chewing guild of insects was demonstrated for such SLCs as flavonoid aglycones [8] and terpenoids [10].

During the last two decades, a number of studies have built up, showing that the effect of host plant resistance is delivered through the food chain via herbivores to next participants of the chain (reviewed in [12]). This phenomenon which was proposed by several groups of researchers showed the direct effect of highly-reactive toxic plant molecules on entomopathogens (consumers of the second level) within the midgut of insects (consumer of the first level) [13–14]. However, during the last few years, a number of entomologists have shown that the quality of food consumed by defoliators can affect one further parameter of the herbivore organism. It is innate immunity, which plays the most important role in an insect—the pathogen interaction which offers effective protection against a wide range of parasites [15–19]. The innate immunity of insects consists of the structures executing barrier functions (cuticle, peritrophic envelope), and the reactions of cellular and humoral immunity (e.g. encapsulation, phagocytosis, antimicrobial peptides, etc.). The encapsulation of an invader followed by its melanization is an effective response of insects to internal parasites such as parasitoids or entomopathogens. The formation of capsules requires the aggregation of plasmatocytes and granulocytes around the invader. The phenoloxidase (PO) cascade takes part in the melanization of hemocytes attached to the surface of the parasite [20]. The baseline status of insects’ innate immunity depends on the contents of nutrients and allelochemicals consumed. In particular, larvae of *Spodoptera littoralis* fed on the protein rich artificial diet, possessed high antimicrobial as well as phenoloxidase activity in hemolymph, and a high encapsulation rate [17]. Ojala et al. [15] have shown that different types of host plants, significantly differing in the profile of allelochemicals, may cause different effect on the encapsulation activity of *Parasemia plantaginis*. Larvae of *Pieris rapae* possessed a different ability to encapsulate the eggs of parasitoids *Cotesia glomerata*, whether they were reared on wild or on cultivated population of cabbage (*Brassica oleracea*) which also sufficiently differed in their chemical profile of allelochemicals [21]. However, in the studies conducted on *Grammia incorrupta* with a separate group of allelochemicals, the authors found neither the effect of ingested pyrrolizidine alkaloids [22] or iridoid glycosides [23] on the melanization of artificially injected particles into insects hemocoel This means that separate components of the plant defense have different effects on insects’ immunity as compared with more complicated constitutive/induced defensive reactions of the whole plant organism.

In our earlier study, we clearly demonstrated that some phenolics, mainly surface flavonoid aglycones, were involved in the induction of silver birch *Betula pendula* Roth resistance after the severe defoliation of trees by gypsy moth larvae [18–19]. These changes in the leaf chemistry of defoliated host plants were associated with a significant delay in larvae development, a decrease in pupal mass of females, and a decrease in the survival rate of insects fed on the leaves of those plants [18–19]. Insect immunity was also affected by plant induced resistance [18–19]. We surmised that surface lipophilic compounds are one of the main phytochemicals involved in silver birch resistance against folivorous insects. Thus, we hypothesize that the SLCs of silver birch leaves are responsible for, (1) the negative effect of host plant leaves on gypsy moth fitness, and (2) the effect on insects innate immunity when plant induced defense occurs. To test this hypothesis, we altered the SLCs concentrations in both natural and artificial diets and following studied treated insects. The experiments were carried out in the summers of 2010–2011. For this study, we used silver birch *Betula pendula* Roth. (*Betulaceae*)—gypsy moth *Lymantria dispar* L. (Lepidoptera: Lymantriidae) system. Gypsy moth is a widespread forest defoliator possessing the ability to form outbreaks across large areas. Silver birch is the preferable host plant of *L*. *dispar* in the Western Siberia region—the place where the experiment was performed.

## Methods and Materials

### Study species

Egg masses of *L*. *dispar* used in the experiment were collected in 2009 and 2010 during an outbreak in *B*. *pendula* stands in Trans Ural region (56° 30′ N 61° 40′ E). Egg masses were held at 4°C until deployment at the beginning of the experiments [18].

No permits for a field collection were required for this study, since the national forests in Russia are freely accessible. No protected species were sampled. Birch trees used for the feeding of insects in this study were grown on the territory owned by the Russian Academy of Sciences.

### Natural diet assay

In 2010, we carried out the no-choice experiment where we modified the presence of SLCs in consumed leaves during insects’ period of growth, according to the procedure reported by Lahtinen et al. [8]. We used a brief interval of 10 secs for washing leaves with ethanol 96% followed by a 5 secs interval for rewashing them with water before the leaves were consumed by insects. We grew two cohorts of insects: one that consumed the birch leaves with a natural (“high”) concentration of SLCs during whole larval stage (control, leaves were washed with distilled water); a second that consumed leaves with low SLCs content. Insects were reared in containers on cut birch branches in laboratory conditions at 23°C under a regime of natural daylight. After washing, branches were dried at room temperature and moved to containers with insects. The fresh branches were put into tubes filled with water and sealed with parafilm to maintain the cell turgor in the leaves. The procedure was repeated every other day during larvae rearing. We used the same tree as well as same type of shoots in a single feeding procedure which allowed for control of the effects in the variation in the content of the secondary compounds between and within trees. We also used different tree individuals between different feedings to control the effect of previous cutting on tree chemistry. Altogether, 1000 insects were used in this experiment. Half of those were reared on control leaves and the other half on ethanol treated leaves. Two hundred and fifty larvae from both cohorts were used to measure fitness and the other two hundred and fifty larvae were assigned to measure the immune parameters. To control the effectiveness of the approach for decreasing SLCs’ concentration on our birch species, we carried out an additional experiment. In particular, one pooled sample of silver birch leaves (ca.400 leaves) was divided into two equal parts. One part of the leaves was washed with ethanol (the sample Nr1), and another with distilled water (the sample Nr2), as described above. Then, both samples of leaves were dried at room temperature in the shade; ethanol extract, obtained after the washing of the leaves (sample Nr3) was evaporated up to 40% of dry matter and all three samples were sent to the laboratory for chemical analysis to estimate the effectiveness of washing.

### *L*. *dispar* fitness traits

We measured the following parameters: 1) weight of small instar larvae (I-II larval instars, most vulnerable stage associated with negative effect of leaf SLCs); 2) weight of middle instar larvae (III-IV larval instars, to record the maintenance/accumulation of the effect of SLCs); 3) larval development time; 4) pupal weight and 5) the survival rate of insects over a period of whole ontogenesis. The female pupal weight of studied species is positively associated with adult fecundity [24], so in the case of females we measured the potential fecundity. The measurements were made according to the procedure reported by Martemyanov et al. [19].

### *L*. *dispar* immune traits

When larvae assigned to have their immune activity measured reached the fourth instar, they were sampled for immune assays. Encapsulation rates, the phenoloxidase activity of hemocytes-free hemolymph and the total hemocytes count (THC) in the hemolymph were estimated according to the procedure reported by Martemyanov and coauthors [18–19, 25]. Briefly, to collect hemolymph, caterpillars were pierced with a thin needle under penultimate proleg. THC was immediately measured with a hemocytometer and the number of hemocytes per 1 ml of hemolymph was recorded. The PO activity of hemocytes-free hemolymph was measured using L-dopa (concentration 2mg/ml solution) as a substrate. The PO activity of samples is presented in terms of the units of transmission density (ΔA) of the incubation mixture during the reaction per 1 min and 1mg of protein. Hemolymph protein levels were measured by the Bradford assay, with a standard curve created from a bovine serum albumin standard. The encapsulation response was measured as the degree of melanization of a nylon monofilament implant that was inserted into the hemocoel of studied larvae. This is a commonly used technique to measure the strength of immunity in insects [26] and is associated with resistance against real pathogens in moths [27]. The degree of the melanization was quantified using Image Pro software by first measuring the coloration—gray value (g.v.) of all areas on each implant, and then comparing these values with that of an unused implant.

### Artificial diet assay

This part of the study was conducted in 2011. We used pooled extract of leaf SLCs, obtained after the washing of leaves, which was used for feeding of treated insects in 2010. We carried out the dose-modified artificial diet-incorporated assay. Newly hatched *L*. *dispar* larvae were reared in Petri dishes (25 larvae per dish) on an artificial diet according to the procedure reported by Ilyinykh [28]. The following concentrations of extract in the diet were used: 0.5%, 1.9%, and 3.8%. The same volume of ethanol/distilled water was also added in the diets to control the effect of the solvent. These concentrations were chosen on the basis of the content of total flavonoid aglycones in birch leaves—representative of SLCs and highly toxic for Lepidopterans’ larvae [8], which are involved in the silver birch induced chemical response after defoliation by gypsy moth larvae [18–19]. At an extract concentration of up to1.9%, the content of flavonoid aglycones in artificial diet was equal to its content in the leaves of native plants; at an extract concentration of 0.5%, the content of flavonoid aglycones was markedly less than that in birch leaves; and at an extract concentration of 3.8%, it was equal to its content in the leaves of birches with induced chemical defense [19]. We used 50 larvae per each treatment. Larvae mass was estimated on the seventh day of feeding, and the survival rate on the ninth day of feeding.

### Chemical analysis

Well ground air-dried leaves of samples Nrs 1 (washed by ethanol) and 2 (washed by water) obtained in an additional control experiment were used for the chemical analysis. Two grams of leaf powder were thrice extracted with 25 ml of 70% ethanol at boiling point over a period of 30min. After centrifuging (10,000xg, 15 min) the extracts of samples Nrs 1 and 2, they were used together with evaporated sample Nr 3 for the further estimation of the content of both glycosides and aglycones of flavonoids used as “markers” of hydrophilic and lipophilic compounds respectively. Sample Nr 3 was also analyzed in more detail: the content of chemical groups of compounds and the chemical profile of flavonoid aglycones were identified. HPLC/DAD was used to estimate the chromatographic profile of low-molecular phenolics and their total content in samples Nr 1, 2, and 3. HPLC/MS was additionally used for the identification of lipophilic flavonoid aglycones in sample Nr 3. Methods GC and GC/MS were used for the identification and detection of the main chemical groups of compounds of sample Nr 3. Prior to its analysis, sample Nr 3 was trimethylsilylated with a mixture of bis(trimethylsilyl)trifluoroacetamide and trimethylchlorosilane (BSTFA + TMCS, 99:1), (Sigma-Aldrich) [29].

HPLC/DAD analysis was performed with an Agilent LC 1100 chromatograph equipped with a quaternary pump, an autosampler and a diode array detector. The chromatographic conditions were as follows: ZORBAX Eclipse XBD-C8 column (4.6x150 mm, 5-μm particles); mobile phase consisting of methanol/0.1% (v/v) TFA in H_2_O gradient; the methanol percentage in the gradient was 0–100% (0–20 min), 100% methanol (20–25 min); flow rate was 0.8 ml/min; injection volume was 2 μl; the detection was performed simultaneously at four wavelengths—254, 280, 320 and 360 nm. Quantification of the flavonoid glycosides of *B*. *pendula* in the samples Nrs 1 and 2 was performed using the standard compound rutin (Fluka); flavonoid aglycones of *B*. *pendula* in samples Nrs1, 2 and 3 were quantified using quercetin (Sigma-Aldrich) as the standard (S1 Table and Table 1). Quercetin was used to make an approximate comparison between this study and the studies of Martemyanov et al. [18–19]; these being the background for the present study.

**Table 1:** Content of individual flavonoid aglycones washed from *Betula pendula* leaves surface.

Peak number^a^	Retention time (min)	Absorption maxima (nm)	Molecular weight	Name ^a^	Content in dry matter of leaf flavonoids, in terms of quercetin (%)
1	16.03	256, 372	**286**	Kaempferol	0.1
2	16.34	274, 334	**300**	Tetrahydroxyflavone methyl ether	0.3
3	16.44	268, 334	**270**	Apigenin	0.2
4	16.63	256, 354	**360**	Not identified flavonoid	0.2
5	16.78	266, 346	**330**	Pentahydroxyflavone dimethyl ether	0.1
6	17.14	276, 346	**344**	Pentahydroxyflavone trimethyl ether	0.2
7	17.24	268, 348	**314**	Tetrahydroxyflavone dimethyl ether	0.1
8	17.54	274, 346	**374**	Not identified flavonoid	0.1
9+10^**c**^	18.22	274, 334	**314, 284**	Tetrahydroxyflavone dimethyl ether, not identified flavonoid	2.2
11	18.65	270, 338	**344**	Pentahydroxyflavone trimethyl ether	0.6
12	19.92	266, 330	**298**	Apigenin dimethyl ether	0.1
Total					4.2

^a^ Numbers of peaks correspond with chromatographic profile in supplementary material.

^b^ For tetrahydroxyflavone—OH-groups in positions 5, 7, 3' и 4'. For pentahydroxyflavone—OH-groups in positions 5, 7, 3', 4' и 5'.

^c^ Peaks of compound 9 and 10 are not separated, ratio of the compounds 9 and 10 determined by the HPLC/MS and is ~ 20:1.

Flavonoid aglycones of sample Nr 3 were also quantified using a UV- spectrophotometer (Cary 5000, Varian), using apigenin (Sigma-Aldrich) as the standard.

HPLC/MS analysis was performed using an Agilent LC 1200 chromatograph equipped with a quaternary pump, a vacuum degasser, an autosampler, a thermostatted column compartment, a diode array detector (Agilent Technologies) and a mass spectrometry micrOTOF-Q (Bruker) detector. Chromatographic conditions were as follows: Zorbax XDB-C8 (2.1x50 mm, 3.5-μm particles); mobile phase consisting of methanol/0.1% (v/v) HCOOH in H_2_O gradient, the methanol percentage in the gradient was 50% (5 min), 50–100% methanol (5–25 min), and 100% (25–35 min); and the flow rate was 0.2 ml/min. The MS was performed with electrospray ionization and an atmospheric pressure (API-ES) source. MS conditions were as follows: Vcap 4000 V; nebulizer pressure 1.6 bar; drying gas (N_2_) flow 8 l/min; drying gas temperature 230°C; and negative and positive scans in the range m/z = 100–1000.

The GC analyses were performed on an Agilent 6890 chromatograph with a flame ionization detector. The column was HP-5 with a length of 30 m and an inner diameter of 0.32 mm, and the film thickness was 0.25 micron in the stationary phase. Since helium was used the carrier gas, at a flow rate of 1.0 ml/min, the sample injection was performed splitless. The temperature of the injector and detector was 280°C, and the column temperature varied according to the following program: 50°C was maintained for 2 minutes, and then increased to 280°C at a speed 10°C/min and maintained at this temperature for 40 min. The contents of the components were calculated by internal normalization without using correction factors.

GC/MS analysis was performed on an Agilent 6890 chromatograph with MS detector HP5975 model. The column was HP-5MS with a length of 30 m and an inner diameter of 0.25 mm, and film thickness being 0.25 micron in the stationary phase. As the carrier gas used helium, at a flow rate 1.0 ml/min, the sample injection was performed splitless. The inlet line and the MS source were held at 280 and 230°C, respectively, and the column temperature program was changed similar to that described above. To identify compounds, NIST 02 Mass Spectral database was used.

### Statistical analysis

Analyses were performed with STATISTICA 6.0 statistical software. The data of insect fitness as well as immunological parameters were tested for normality with a Kolmogorov–Smirnov’s test. None of the traits’ data were differed significantly from a normal distribution except for larval PO activity in hemolymph and the duration of the larval stage. For this two parameters we log_10_-transformed the variables before the analysis and again checked the normality using Kolmogorov–Smirnov’s test. Since log_10_-transformation resulted in a normal distribution of variables, we used a two-way ANOVA to test all immunological data, pupal weight, and larval development time, using the treatment and sex of insects as categorical predictors. We did not use nested design statistics because larvae in each of the containers were fed by leaves of the same tree within each meal, i.e. the factor “tree” was excluded. The comparison of each case with other cases was made by Post hoc Fisher LSD procedure. Larval mass was compared by one-way ANOVA because we did not identify the sex of the young larvae. The significance of insects’ survival rate was analyzed by one-way ANOVA. Prior to analysis, all data in percentages were arcsine of square root-transformed.

## Results

### Chemical analysis of washed leaves and the ethanol extract used for artificial diet assay

In our study using HPLC/DAD, we have shown that the approach of washing SLCs by ethanol decreases the concentration of SLCs in treated leaves while no changes of hydrophilic compounds’ concentration (like flavonoids glycosides) are recorded in the treated leaves (S1 Table, S1a,b 2 Fig) In particular, the concentration of lipophilic flavonoids (used as the marker of SLCs, accumulated in leaf glandular trichomes) was reduced ten-fold. By GC/MS, we found that besides lipophilic flavonoids from leaf surface, following groups of compounds were also washed off: triterpenoids and sterols, aliphatic hydrocarbons, fatty and phenolic acids derivatives (Table 2). Within triterpenoids, we identified the derivatives of paperific acids—tetracyclic triterpenoids containing a dammarane type of skeleton, which is common for this species of birch [10, 30]. We also individually identified the flavonoid aglycones (Table 1). The total content of flavonoids aglycones in washed sample Nr 3 was 4.2% in terms of quercetin. The maximal absorption in UV–spectra of the major compound was 335 ± 3 nm that corresponded with the flavonoids containing an apigenin skeleton. In this connection, we also determined the concentration of flavonoids by UV-spectrophotometry in terms of the apegenin that was different compared to that determined by HPLC/DAD using quercetin standard (5.4% and 4.2% correspondingly).

**Table 2:** General content of lipophilic compounds washed by 96% ethanol from surface of *Betula pendula* leaves.

Nr	Name of compounds or chemical groups	Content in dry matter of leaf SLCs,%
1	Triterpenoids and sterols	71
2	Aliphatic hydrocarbons, fatty and phenolic acids derivatives	14
3	Flavonoids	5^a^
4	Not identified compounds	10

^a^The concentration of flavonoids is little differed from the concentration, presented in Table 1. This difference is mediated by using of different methods of chromatography (GC and GC/MS in this table vs. HPLC/DAD in Table 1)

### Effect of SLCs on insects’ fitness in the natural diet assay

**Fig 1:**
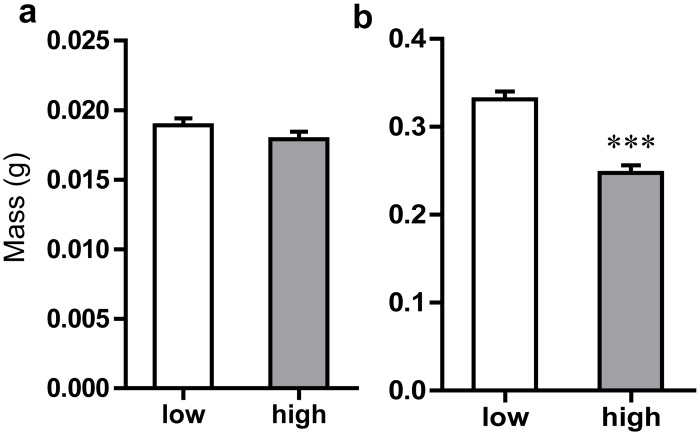
Effect of *Betula pendula* leaf surface lipophilic compounds on *Lymantria dispar* larval weight. The weight (mean ±SE) of young (a) and middle (b) instar larvae reared on leaves with low and high concentrations of surface lipophilic compounds is presented. The data were compared using a one-way ANOVA. Asterisk means the significant differences between bars (at *P*<0.05).

**Fig 2:**
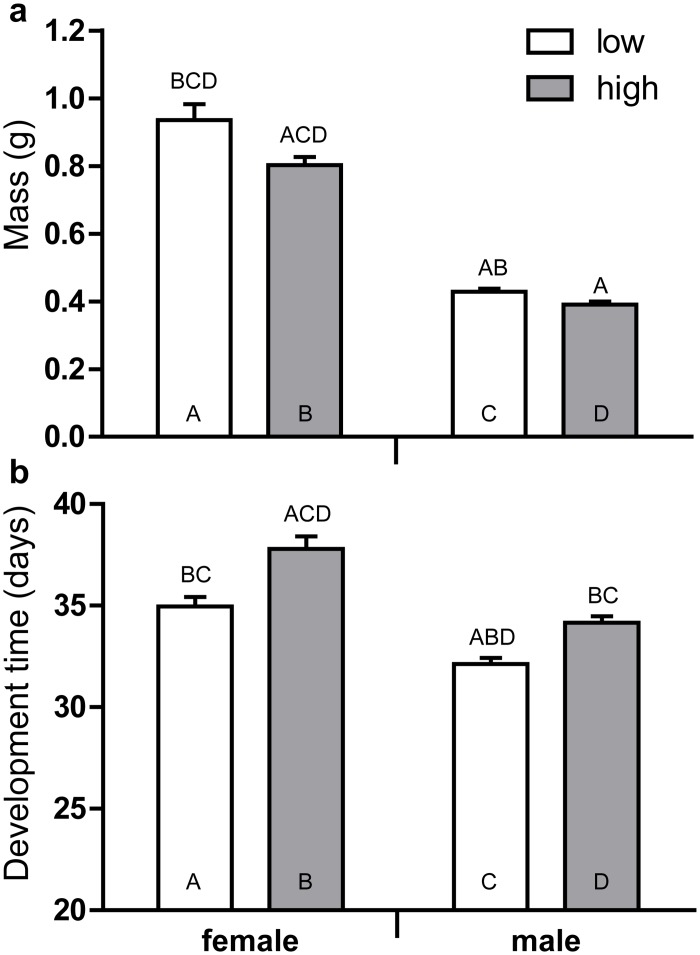
Effect of *Betula pendula* leaf surface lipophilic compounds on pupal weight and larval stage duration. The weight (mean ±SE) of pupae (a) and duration (mean ±SE) of larvae stage (b) of *Lymantria dispar* reared on *Betula pendula* leaves with low and high concentrations of surface lipophilic compounds is presented. The data were pair-wise compared using a post-hoc Fisher LSD procedure. The letters above the bar mean the significant differences (at *P*<0.05) to be compared with the bars abbreviated by the same letters within the bar.

**Fig 3:**
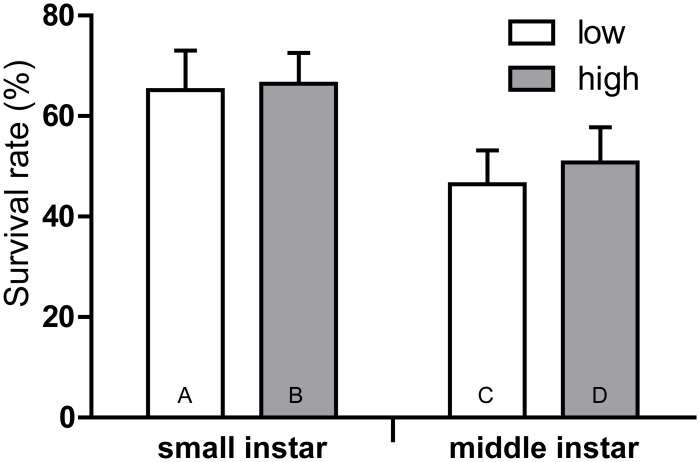
Survival rate of larvae reared on *Betula pendula* leaves with different concentrations of surface lipophilic compounds. Bars are mean ±SE. The data were pair-wise compared using a post-hoc Fisher LSD procedure. The letters above the bar mean the significant differences (at *P*<0.05) to be compared with the bars abbreviated by the same letters within the bar.

High SLCs content in birch leaves did not significantly affect early instar larvae weight (*F*_*1*, *32*_ = 2.31, *P* = 0.128, Fig. 1a) but significantly decreased the weight of middle instar larvae (*F*_*1*, *241*_ = 19.047; *P* <0.001, Fig. 1b). Female pupael weight was higher when larvae consumed birch leaves with low SLCs content compared to the consumption of leaves with high SLCs content (Fig. 2a, Table 3). A similar trend was found in males but it was not statistically significant (*P* = 0.078 by Fisher LSD test, Fig. 2a). The larval development rate was increased when larva consumed leaves with low SLCs content, as compared to the consumption of leaves with high concentrations of SLCs (Fig. 2b, Table 4). However, the survival rate of both early and middle instars larvae was the same between compared treatments (Fig. 3).

**Table 3:** Factorial ANOVA results of comparison of pupae weight between females and males reared on *Betula pendula* leaves with low and high concentrations of surface lipophilic compounds.

Effect	df 1	df 2	*F*	*P*
Treatment	1	146	20.36	<0.001
Sex	1	146	586.32	<0.001
Sex*treatment	1	146	6.30	0.013

**Table 4:** Factorial ANOVA results of comparison of larval stage duration between females and males reared on *Betula pendula* leaves with low and high concentrations of surface lipophilic compounds.

Effect	df 1	df 2	*F*	*P*
Treatment	1	150	30.15	>0.001
Sex	1	150	61.21	>0.001
Sex*treatment	1	150	0.18	0.674

### Effect of SLCs on insect immune parameters in the natural diet assay

**Fig 4:**
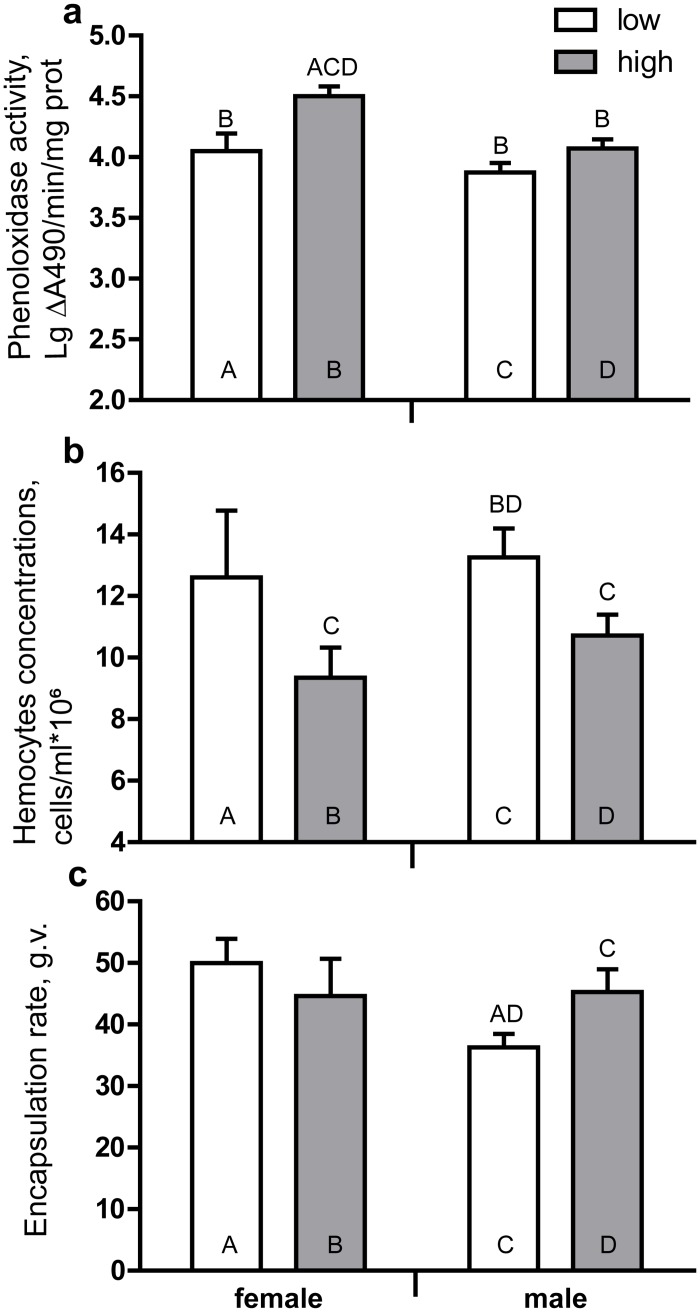
Effect of *Betula pendula* leaf surface lipophilic compounds on larval innate immunity parameters. Activity (mean ±SE) of phenoloxidase in hemocytes-free hemolymph of hemolymph (a), total hemocyte count (mean ±SE) in hemolymph (b) and encapsulation rate (mean ±SE) of hemolymph (c) of fourth instar *Lymantria dispar* larvae reared on *Betula pendula* leaves with low and high concentrations of surface lipophilic compounds is presented. The data were pair-wise compared by the post hoc Fisher LSD procedure. The letters above the bar mean the significant differences (at *P*<0.05) to be compared with the bars abbreviated by the same letters within the bar.

The activity of PO in the hemocytes-free hemolymph of larvae and TCH were both significantly affected by higher SLCs concentration in leaves (Table 5). The trend was the same for both sexes: THC was higher but PO was lower when larvae were fed with leaves with low SLCs content (Fig. 4a,b). The consumption of leaves with low SLCs content resulted in a decrease of encapsulation rate but only in males’ hemocoel and not in females’ hemocoel (Fig. 4c).

**Table 5:** Factorial ANOVA results of comparison of larval immune parameters between females and males reared on *Betula pendula* leaves with low and high concentrations of surface lipophilic compounds.

Effects	df 1	df 2	*F*	*P*
^a^Treatment	1	119	7.65	**0.007**
^a^Sex	1	119	6.65	**0.011**
^a^Sex*treatment		119	0.32	0.281
^b^Treatment		122	4.84	**0.030**
^b^Sex		122	0.590	0.444
^b^Sex*treatment		122	0.072	0.790
^c^Treatment	1	111	0.170	0.681
^c^Sex	1	111	2.125	0.149
^c^Sex*treatment	1	111	2.569	0.112

^a^The activity of phenoloxidase in hemocytes-free hemolymph

^b^Total haemocytes count

^c^Encapsulation rate.

### Effect of SLCs on insects’ fitness in the artificial diet assay

**Fig 5:**
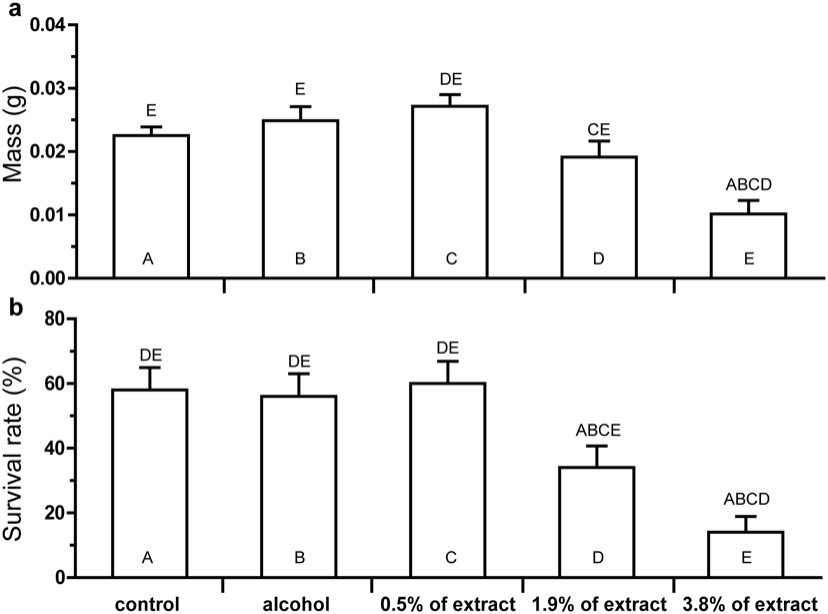
Effect of *Betula pendula* leaf surface ethanol extract on *Lymantria dispar* fitness. Weight (mean ±SE) (a) and survival rate (mean ±SE) (b) of *Lymantria dispar* larvae reared on artificial diet with different concentrations of leaf surface extract is presented. The data were pair-wise compared by post hoc Fisher LSD procedure. The letters above the bar mean the significant differences (at *P*<0.05) to be compared with the bars abbreviated by the same letters within bar.

Neither ethanol alone nor the extract added to the artificial diet at low concentration affected larval weight or the mortality rate. Meanwhile, the higher concentrations of ethanol extract (1.9% and 3.8%) led to a dose-dependent decrease of larvae weight (*r* = -0.992; *P* = 0.079) as well as survival rate (*r* = -0.999; *P* = 0.017) rate (Fig. 5a,b).

## Discussion

Our study shows that silver birch leaf SLCs negatively affect the fitness of *L*. *dispar*. The main chemical groups of phytochemicals in silver birch were found to be triterpenoids, sterols, aliphatic hydrocarbons, fatty and phenolic acid derivatives and flavonoid aglycones which was also shown in other studies on the same [9,10] and closely related species [8]. Fed on leaves with low SLCs concentrations led to a high rate of larval development and an increase in the weight of middle instar larvae. According to pupal weight data, the effect was more significant for females than for males. The high SLCs concentration in artificial diet showed a vice-versa effect: a dose-dependent decrease of larvae weight and an increase in the mortality rate. Interestingly, in another study conducted on *Epirrita autumnata* (Lepidoptera: Geometridae) where the effect of SLCs (mainly flavonoids aglycones) was studied, its authors found the same effect of the SLCs of mountain birch on insects [8]. However, in that study SLCs only affected the early instar larvae and did not affect fifth instar larvae. In the present study, we found the reverse picture in the “natural diet” experiment: the negative effect of SLCs accumulated from early instar (only the trend was registered) reached significant differences at middle larval development and remained until pupal stage. Combining this result with those from the artificial died experiment where young larvae were significantly affected by high doses of SLCs, the differences between the two studies could be explained by a higher threshold of sensitivity of *L*. *dispar* young larvae to SLCs concentrations in comparison with that of the young larvae of *E*. *autumnata*. Moreover, we found that *L*. *dipar* females were more sensitive to the consumption of SLCs than males. This result reflects that different insects defoliator species respond to the content of SLCs in leaves in different ways. The sex-specific effect of SLCs on *L*. *dispar* pupae weight could be explained by a different physiology of digestion between both sexes. Particularly, female larvae are affected by protein deficit while males prefer to consume the lipid-rich diet [31]. This effect was caused by the life-history strategies of different sexes of *L*. *dispar*, implying implying a male requirement for lipids due to their flight activity versus a female requirement for proteins due to oogenesis [31]. One further possibility, which could explain such sex-specific effects of SLCs on insect fitness is the developmental dimorphism that exist between juvenile stages of *L*. *dispar*. In particular, females possess one additional instar in comparison to males [32]. Moreover, the relative consumption rate of the last instars of females is higher than males’ consumption in the same instars [31]. Phenols present in SLCs dysplay the ability to precipitate proteins, making them inaccessible for digestion. Consequently, a low SLCs concentration in food allows insects to digest more proteins, and females become more responsive to this process than males. The sex-specific decrease of *L*. *dispar* pupal weight under low-quality food was demonstrated in our previous studies [19, 25]. The higher pupal weight in *L*. *dispar* females is usually positively associated with their fecundity [24], which means that SLCs might negatively affect the amount of offspring in this species and could bring a decline in the population density of the next generation. However, the low SLCs content in washed leaves did not significantly affect the survival rate. This result is not in accordance with results obtained in *E*. *autumnata* study where researchers clearly demonstrated the direct correlation between the content of surface flavonoids (components of SLCs) and the mortality rate of first instar larvae [8]. There are several possibilities that can explain these differences. The first is that SLCs from different birch species (*B*. *pendula* vs. *B*. *pubescens*) produce different effects—even on the same species of defoliator [9]. The second is that the larvae of *L*. *dispar* are less sensitive to the toxic effects of SLCs, compared *E*. *autumnata* larvae. Finally, there could be a toxic threshold for SLCs concentrations in food for *L*. *dispar* larvae which corresponds with SLCs concentrations in native leaves. This final speculation is confirmed by the result of our artificial diet experiment: the addition of SLCs at a concentration of 0.5% did not change the larval survival rate, whereas higher concentrations significantly increased the mortality rate (Fig. 5b).

Interestingly, low SLCs content in consumed food positively affects the THC in hemolymph of *L*. *dispar* larvae. It is known that some types of SLCs such as flavonoid aglycones possessed cytotoxic activity that was shown in a tumor cells line [33]. However, several studies carried out on invertebrates show that flavonoid aglycones do not reach the hemocoel because of the glycosilation of toxic molecules within the midgut [34–38]. Thus, we suppose that the effect of SLCs is more complex than a direct cytotoxic activity, but rather by possibly indirectly affecting hemocytes proliferation or differentiation. The observed increase in THC in plasma of insects reared under low SLCs content is a possible consequence of the decreased costs of the glycosilation (or other ways of detoxification) of SLCs in insects midgut.

Surprisingly, PO activity in the hemolymph was increased when larvae consumed leaves with high SLCs concentrations. It is possibly because SLCs lead to minor, but repairable, damage to the midgut epithelium cells, sufficient to elicit immune reactions in the hemolymph through signaling molecules generation [39], but not enough for the irreversible destruction of gut tissue. It is also important to note that basic PO activity was sex-dependent. This finding confirms earlier work summarizing the sex-mediated differences in PO activity within insects (compared in [40]) and emphasizes the importance of sex identification during immune assay even at juvenile stage of insects.

We demonstrated the sex-dependent effect of leaves SLCs content on the encapsulation rate of larvae. The differences in the basic status of immune parameters between the sexes of invertebrates have been demonstrated in many studies [27, 41]. However, the induction of different immune responses in males/females mediated by host plant quality has been little studied. In our recent work, we demonstrated that the delayed induced response of trees result in an increase in the encapsulation rate of female larvae of *L*. *dispar* fed on defoliated birches but does not affect the same immune parameter for males [25]. We propose that such sex-specific inductions might be mediated by food-induced hormonal dependent sexual dimorphism in immune function. In this study, we extracted lipophilic compounds from the leaf surface. Thus the concentration of lipophilic precursors of insects’ hormones (ecdysteroids and juvenile hormone) such as cholesterols/fatty acids [42–43] was possibly lower in the food of those larvae which consumed food after alcohol treatment. It has been shown that the both types of hormones mediate insect immunity [44–46] and they might provide sex-specific modulation of the immune function of males/females that has been shown in other species [47, 41].

In conclusion, our study has clearly demonstrated that leaf surface lipophilic compounds of silver birch affect both insects’ fitness as well as basic status of their innate immunity. Thus, the concentration of these compounds in the leaves of silver birch determines the level of constitutive or induced defense of tree against gypsy moth. However, the effect of SLCs on larvae innate immunity differs from the generalized induced chemical defense of silver birch [18–19] where concentrations of SLCs are significantly higher compared with native trees [10, 18–19]. This fact reveals that not only SLCs but other classes of phytochemicals or even nutrients involved in plant induced chemical defense are also important for affecting the state of larval immunity. Our study also demonstrates that some immune parameters of females and males even at a juvenile stage are differently affected by food quality. This fact must be taken into account in ecological immunology studies.

## Supporting Information

S1 Fig The chromatograms of flavonoids extracted from silver birch leaves washed with water (A) and ethanol (B). (TIF)

S2 Fig The chromatogram of washed ethanol extract from silver birch leaves surface. (TIF)

S1 Table The flavonoids content in leaves after its washing with 96% ethanol (sample Nr1) and water (sample Nr 2). (DOC)
