# Supplementary figures and images for: Leaf Surface Lipophilic Compounds as One of the Factors of Silver Birch Chemical Defense against Larvae of Gypsy Moth
Source: PLoS One. 2015 Mar 27;10(3):e0121917. doi: 10.1371/journal.pone.0121917 (PMC4376524; doi:10.1371/journal.pone.0121917)

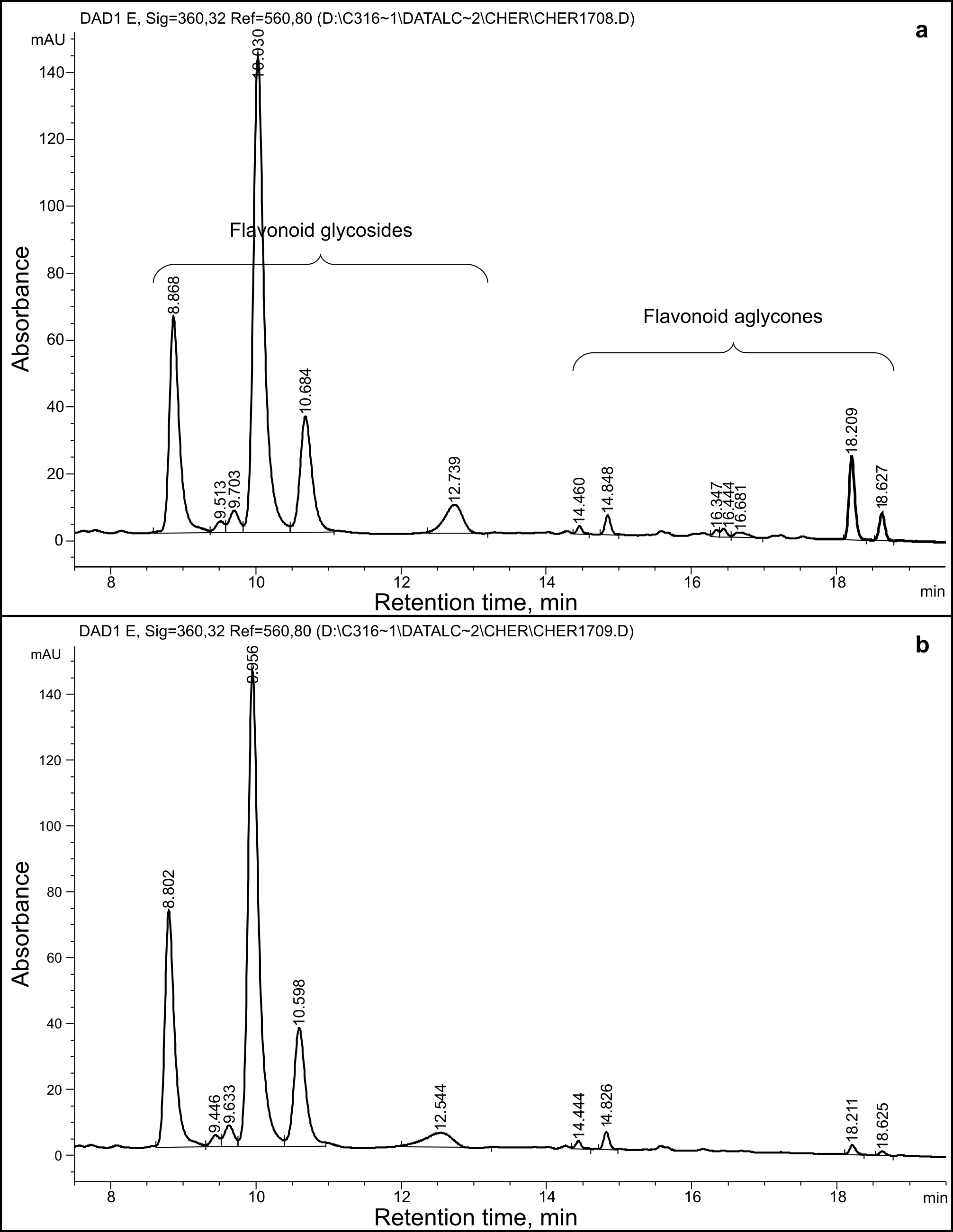

Supplement: S1 Fig — (TIF) [file pone.0121917.s001.tif]

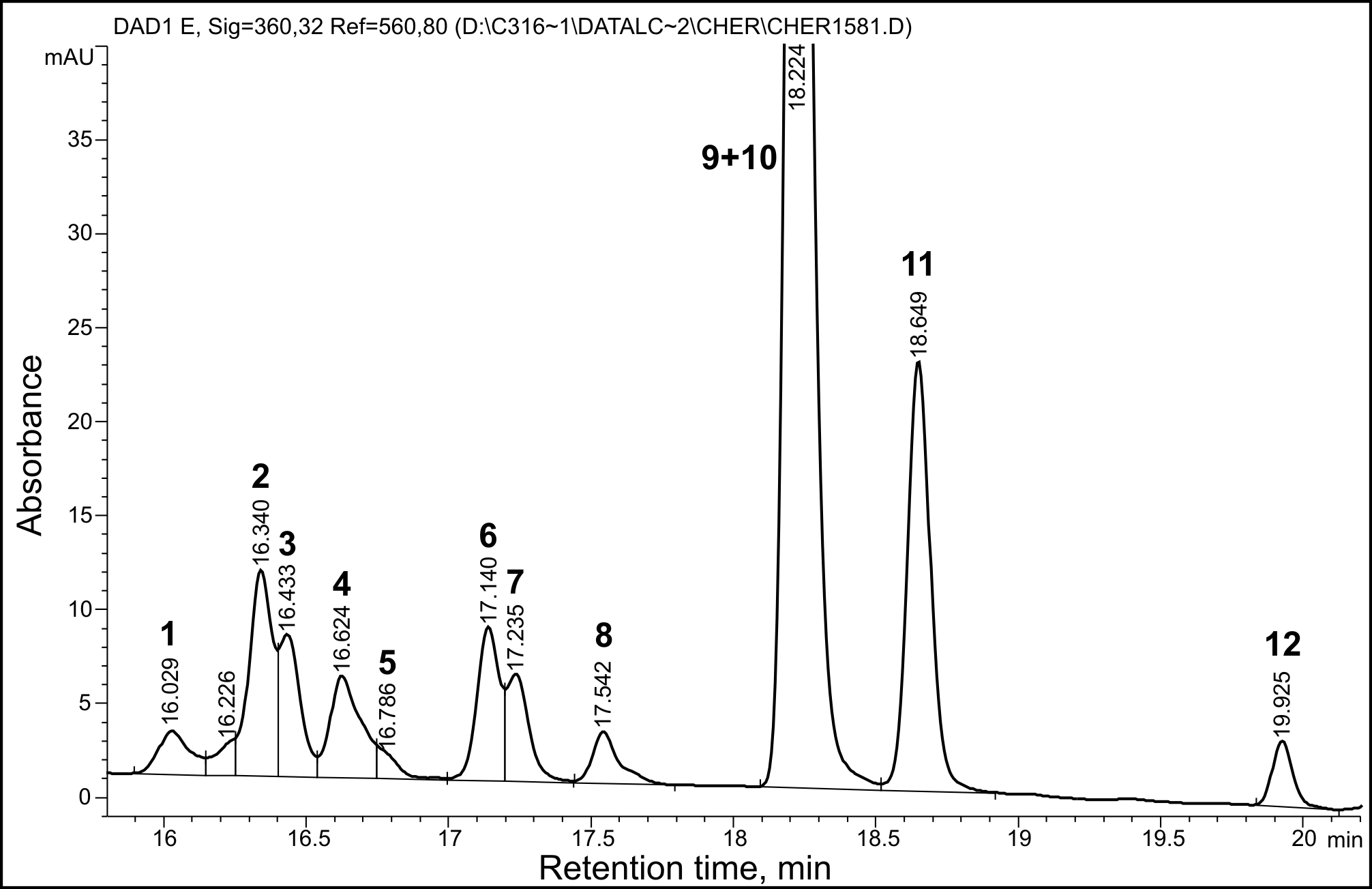

Supplement: S2 Fig — (TIF) [file pone.0121917.s002.tif]
