# Supplementary material for: Leaf Surface Lipophilic Compounds as One of the Factors of Silver Birch Chemical Defense against Larvae of Gypsy Moth
Source: PLoS One. 2015 Mar 27;10(3):e0121917. doi: 10.1371/journal.pone.0121917 (PMC4376524; doi:10.1371/journal.pone.0121917)
Supplement: S1 Table — (DOC) [file pone.0121917.s003.doc]

**S1Table.** The flavonoids content in leaves after its washing with 96% ethanol (sample Nr1) and water (sample Nr 2)

| Samples | The flavonoid concentrations, mg g^-1^ leaf dry weight | |
| --- | --- | --- |
|  | Flavonoid glycosides | Flavonoid aglycones |
| 1 | 24.6 | 0.2 |
| 2 | 23.7 | 1.8 |
